# Effect of DMSO Addition on the Hexagonal Phase of the System Triton X/Water

**DOI:** 10.1021/acs.langmuir.4c01937

**Published:** 2024-10-11

**Authors:** Leila T. Thieghi, Sarah I.P.M.N. Alves

**Affiliations:** Instituto de Ciências Ambientais, Químicas e Farmacêuticas, Universidade Federal de São Paulo, Diadema, SP 09913-030, Brasil

## Abstract

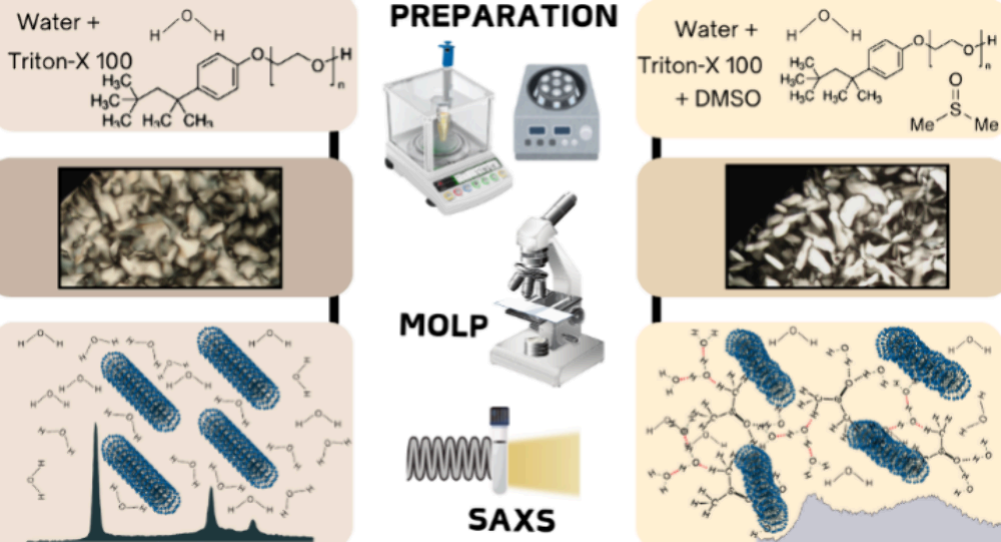

This research studied the role of DMSO in a binary system of Triton X and water in the hexagonal mesophase. One effect of DMSO addition, determined using polarized optical microscopy and small-angle X-ray scattering measurements, is to promote a decrease in the hexagonal to isotropic phase transition temperature, *T*_H-ISO_, decreasing the range of temperatures of the hexagonal phase until the hexagonal phase completely disappears when DMSO is added up to 5.0 mol %. The periodicity and the lattice parameter of the hexagonal arrangement, calculated as a function of DMSO concentration, slight increased due to the insertion of DMSO molecules in the water region, causing a greater distance between the cylindrical micelles, while the radius of the apolar domains kept constant at 22 (1) Å.

## Introduction

Dimethyl sulfoxide (C_2_H_6_OS), DMSO, is a polar aprotic solvent miscible in numerous organic solvents, as well as water, and that dissolves both polar and nonpolar compounds. Due to this property, this compound is largely utilized as a solvent known for its biological and therapeutic properties. It possesses an excellent ability to penetrate membranes and is a good instrument for carrying various compounds through the bloodstream. For this reason, it is interesting to study its addition in a lyotropic liquid crystal, making it possible for some substances not soluble in water to be solubilized in DMSO before they are added to the system. Some works were done about the influence of DMSO on the structure of lamellar lipid membranes:^1−4^ depending on DMSO concentration, [DMSO], its addition makes the membrane significantly floppy, changes the periodicity, and modifies the chain-melting phase transition temperature of membranes; if DMSO is added in low concentrations in membranes in a hexagonal II mesophase, then it stabilizes the mesophase.^5^ It is known that DMSO/water solutions have chemical and physical properties that are quite unexpected for an ideal solution,^6,7^ and this deviation is best seen if these properties are plotted against the mole fraction. Engel et al.^8^ studied DMSO/water mixtures by X-ray absorption and emission spectroscopy and inelastic X-ray scattering and demonstrated that the interaction between DMSO and water is strong: even in water-rich solutions, DMSO molecules distort the hydrogen bond network of water; already at 75% by volume (43% by mole) of DMSO, the interaction between the molecules has a huge effect on the spectra, suggesting that clustering occurs. A research group in India studied the effect of DMSO as a cosolvent in papers about physicochemical properties responsible for the micellization process of anionic, cationic, and nonionic surfactants in water–DMSO mixtures:^9,10^ increasing the concentration of the cosolvent (DMSO) in the water–DMSO mixture, there is a decrease in cohesive force, and surfactant molecules are more soluble in a mixed solvent, resulting in a less favorable micellization process and an increase in critical micellar concentration.

Lyotropic liquid crystals, formed by micelles (aggregates of amphiphilic molecules in a solvent), have been utilized in different applications due to their unique characteristics such as morphologies, hydrophilic and hydrophobic domains, high internal interfacial surface area, and the possibility of phase transitions depending on temperature and concentration. Some of the applications are in drug delivery,^11−13^ templating to create novel nanoscale organic and inorganic materials,^14−16^ microreactors,^17^ and more. No works were found about the DMSO addition in lyotropic direct hexagonal phases, and its effect is not known. A possibility of this addition is modulating the characteristic distances or the region of existence of the hexagonal mesophase, which can be interesting for various applications cited above.

Triton X-100 (Triton X, C_14_H_22_O (C_2_H_4_O)_9.5_) is a nonionic surfactant that has a hydrophilic poly(ethylene oxide) chain and a hydrophobic hydrocarbon chain with an aromatic group in the middle and is largely used in biology. The binary system Triton X-100 and water is a well-known lyotropic system that exhibits three main liquid crystalline mesophases: micellar,^18,19^ lamellar, and hexagonal.^20−22^ Evidence of phase transitions in this binary system has been reported since 1948.^23^ The lyotropic liquid crystalline mesophases depend on the relative concentration of compounds and temperature.^24^ Lamellar and hexagonal mesophases exhibit a certain order,^25^ and the hexagonal one is more ordered than the lamellar one. In the hexagonal mesophase, the micelles have a cylinder-like shape, and these cylinders are positioned in a 2D hexagonal arrangement; two characteristic dimensions define the hexagonal arrangement: the spacing of the corresponding planes, or periodicity, *d*, and the lattice parameter, *a*.^25^ From small-angle X-ray scattering data, these characteristic dimensions can be obtained from the position (*q*_1_) of the first diffraction peak by
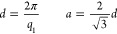


This work investigated the lyotropic liquid crystal in the hexagonal phase doped with DMSO under polarized optical microscopy (POM) and small-angle X-ray scattering (SAXS) techniques. DMSO was added in small quantities in the binary system as doping, and the effects of this doping were studied in the mesophases’ structural dimensions and phase transition temperatures, *T*_H-ISO_.

## Experimental Section

### Materials

Binary mixtures of a nonionic surfactant and water were prepared with Triton X-100 (polyethylene glycol *tert*-octylphenyl ether, molecular mass = 625 g/mol) from Sigma-Aldrich and distilled water. Samples with Triton X-100 concentrations, [Triton X], from 2.3 to 3.3 mol % were prepared by weight. All compounds were mixed in a vortex in an ultrasound bath and centrifuged for homogenization. For doping, DMSO (dimethyl sulfoxide, molecular mass = 78.13 g/mol) from Sigma-Aldrich was directly added to the hexagonal matrix, with [DMSO] up to 5 mol %. All sample concentrations used in this study are shown in the Supporting Information (SI).

### Techniques

#### Polarized Optical Microscopy (POM)

The POM technique provides texture observations of the samples between the crossed polarizers. Each different lyotropic liquid crystal mesophase presents its own texture, so observing them as a function of temperature makes it possible to identify mesophases as a function of the sample’s temperature, *T*. The samples were encapsulated inside microslides with a 0.2 mm thickness sealed with Parafilm, placed in an INSTEC HS1-i hot-stage system, and positioned in a Leitz orthoplan-pol microscope. The samples were heated above the transition temperature from the hexagonal to isotropic phase, *T*_H-ISO_, for some seconds and then cooled to 6 °C at a noncontrolled rate. After being kept at 6 °C for about 1 h, samples were again heated until transition temperature, *T*_H-ISO_, at a controlled rate. Samples with varying temperatures from 6 to 40 °C were observed, and micrographics were taken with a digital camera coupled to the microscope.

#### Small-Angle X-ray Scattering (SAXS)

SAXS experiments were performed in a Xenocs Xeuss SAXS/WAXS system at the Complex Fluid Group, IFUSP, Brazil. It has a GeniX beam delivery system with a Cu anode X-ray tube (λ = 0.15411 nm), two scatterless slits as a collimator, and a two-dimensional Dectris Pilatus 300 K detector, which registers the patterns. The beam delivered to the samples has a square cross section of 0.5 × 0.5 mm. Samples were encapsulated in capillary glass tubes of 1.5 mm diameter. The metal substrate plate was used to ensure accurate heat transfer to and from the sample, and the sample temperature was controlled by a water bath and verified by a hot-stage unit. Measurements were performed at a controlled temperature, *T*, from 6 to 32 °C. The exposure time for SAXS measurements in each sample was 15 min. The circular averaging of the two-dimensional images was performed using the SOLEIL Foxtrot software. The scattering vector modulus defined as *q* = (4π sin θ)/λ, where 2θ is the scattering angle, was provided in the range of 0.02–4.50 Å^–1^. Using simple crystallographic arguments, the hexagonal lattice parameter, *a*, and the spacing of the corresponding planes, *d*, were calculated from the diffraction data. The wider the Bragg peaks, the smaller the coherent scattering domains.^26^ For the hexagonal structures, the position of the three first diffraction peaks in the reciprocal space obeys the relationship ,^25,26^ corresponding to the {100}, {110}, and {200} planes.

## Results and Discussion

This work investigated a lyotropic liquid crystal (Triton X and water) in the hexagonal mesophase doped with DMSO using POM and SAXS techniques. DMSO was added in small quantities in the binary system as doping, and the effects of this doping were studied in the mesophases’ structural dimensions and phase transition temperatures, *T*_H-ISO_.

The results in this section are presented as the following: (1) pure liquid crystal with [Triton X] from 2.3 to 3.3 mol %; (2) liquid crystal with a constant [Triton X] of 2.8 mol % with [DMSO] doping from 0 to 5 mol %; (3) liquid crystal with [Triton X] from 2.8 to 3.3 mol %, with [DMSO] doping from 0 to 5 mol %, increasing proportionally to [Triton X]; (4) liquid crystal with [Triton X] from 2.3 to 2.7 mol %, with [DMSO] doping from 0 to 5 mol %, increasing inversely proportional to [Triton X].

With the aim of understanding the role of each of the compounds, section 1 just characterized a well-known hexagonal region in the phase diagram of the studied binary system; section 2 studied the doping of DMSO up to 5 mol % in a position of the phase diagram corresponding to the largest interval of temperature for existing the hexagonal mesophase, that is, [Triton X] = 2.8 mol %. The water concentration, [H_2_O], in this section varied from 97.2 to 92.2 mol % as [DMSO] varied up to 5 mol %. In the next two sections, [H_2_O] was kept from 92.2 to 97.2 mol % as [DMSO] varied up to 5 mol %, but the [Triton X] also varied: In section 3, [Triton X] varied from 2.8 to 3.3 mol %, with [DMSO] doping up to 5 mol %, increasing proportionally to [Triton X]. In section 4, [Triton X] varied from 2.3 to 2.7 mol %, with [DMSO] doping up to 5 mol %, increasing inversely proportional to [Triton X].

### Pure Liquid Crystal with [Triton X] from 2.3 to 3.3 mol %

**Figure 1:**
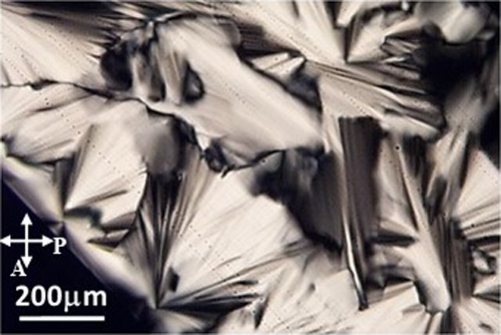
Typical fanlike texture of a hexagonal phase (2.8 mol %) for a 100 μm-thick Triton X/water binary system at a temperature of 25 °C in a polarizing light microscope between crossed polarizers. P and A indicate polarizer and analyzer directions, respectively.

First, phase transition temperatures, *T*_H-ISO_, of a hexagonal region on the phase diagram (from 2.3 to 3.3 mol % of Triton X, Table S1 in the Supporting Information) were well-determined by POM observations in a temperature range from 6 to 40 °C. Optical observations were done at an about 5 °C/min rate, changing to 0.5 °C/min near the transition temperature, *T*_H-ISO_*.* All observed samples presented a hexagonal (Hex) mesophase at low temperatures followed by an isotropic (ISO) mesophase. Figure 1 shows a typical fanlike texture of a hexagonal phase for a 100 μm-thick Triton X-100/water mixture (2.8 mol %) at a temperature of 25 °C in a polarizing light microscope between crossed polarizers. For this [Triton X], the transition temperature was determined as *T*_H-ISO_ = 31.0 (5) °C, which is in good agreement with other authors.^20−22^

**Figure 2:**
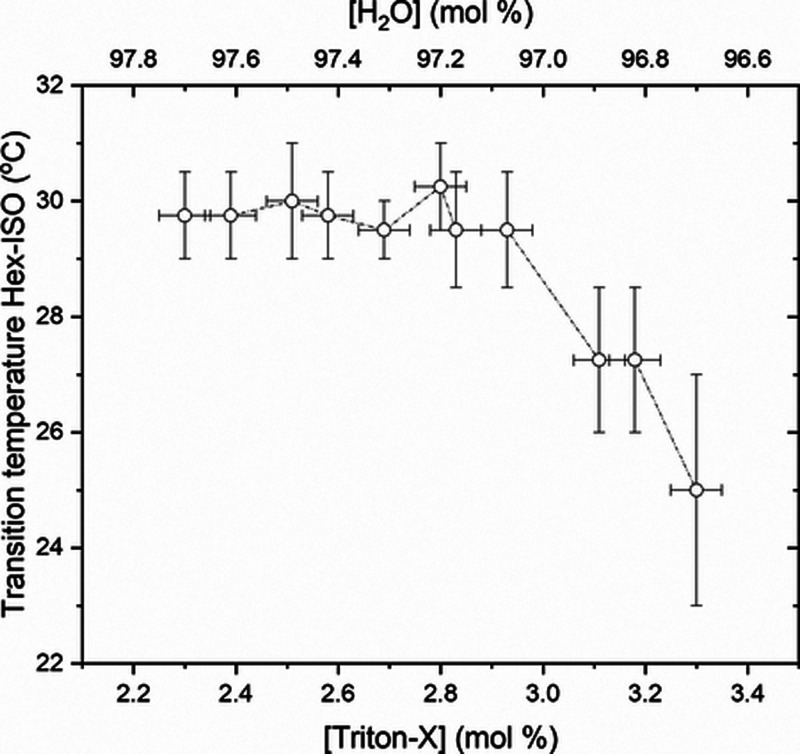
Hex-ISO transition temperatures determined by POM observations for nondoped samples, with [Triton X] from 2.3 to 3.3 mol %.

POM observations were performed for each sample in a range of temperatures from 6 to 40 °C, and Hex-ISO transition temperatures, *T*_H-ISO_, are presented in Figure 2, in good agreement with previous papers.^20,22^Figure 2 shows a transition temperature almost constant from 2.3 to 2.8 mol % [Triton X] followed by a decreasing behavior from 2.8 to 3.3 mol %.

**Figure 3:**
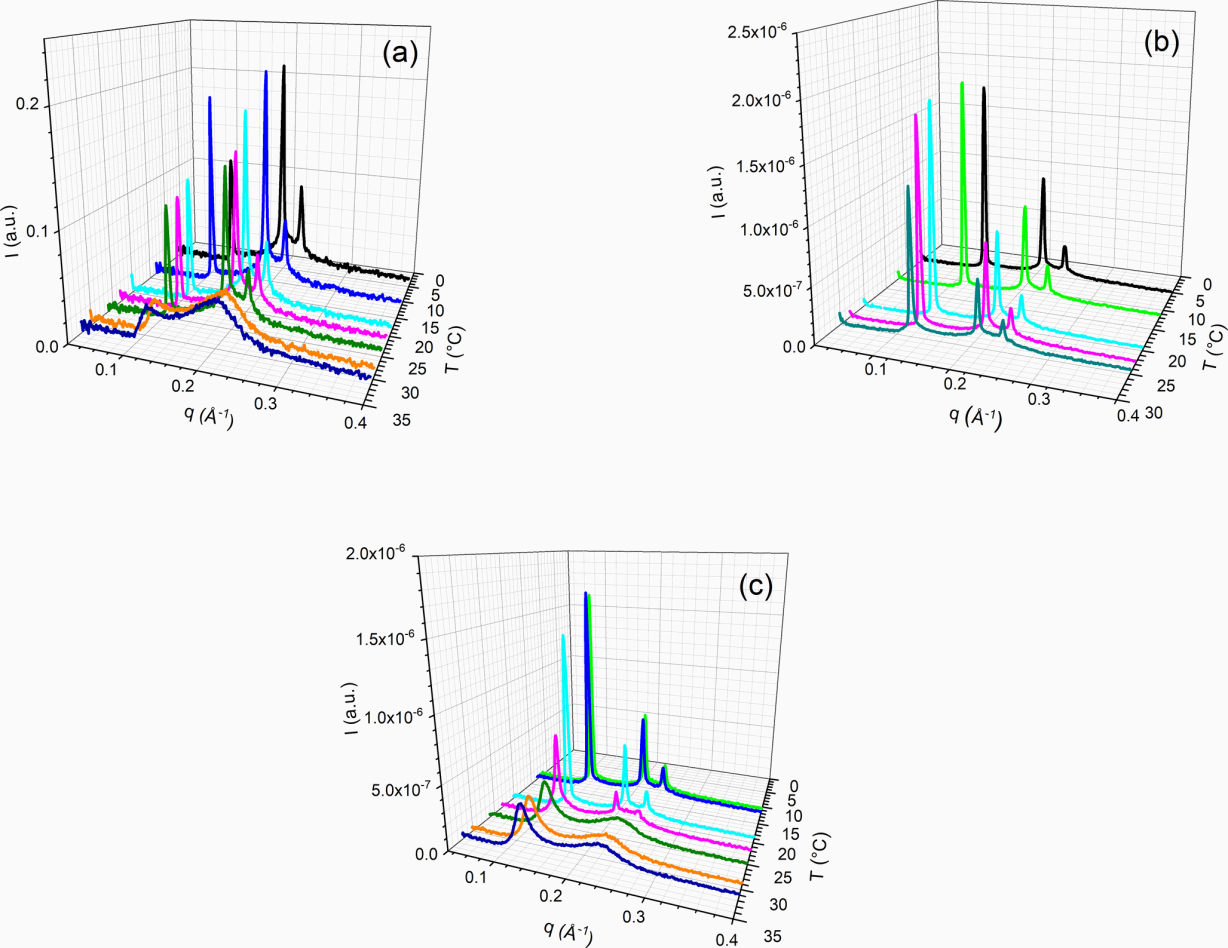
Diffraction peaks obtained at different temperatures for the nondoped samples, with [Triton X] ranging from 2.3 to 3.3 mol %. (a) 2.3, (b) 2.8, and (c) 3.3 mol %.

SAXS measurements were performed through a range of temperatures, and Figure 3 shows some diffractograms for the sample concentrations for [Triton X] from 2.3 to 3.3 mol %. The complete series of diffractograms is shown in Figure S1 in the Supporting Information. All samples lost the hexagonal diffraction peak relation  when near to the transition temperature *T*_H-ISO_ due to the evolution of structure on heating from the hexagonal to isotropic phase. As these peaks are sharp, this small-angle X-ray diffraction data collection accurately calculates the hexagonal phase’s lattice parameter, *a*, and the periodicity, *d*. The relationship shown in Figure 3b confirms the hexagonal phase; with a calculated periodicity *d* = 51.8 (7) Å, the lattice parameter *a* = 59.8 (8) Å at room temperature (23 °C) for the sample 2.8 mol % is in a good agreement with the lattice parameter value of 59.5 Å reported by Ahir et al.^22^ and is 15 Å higher than that founded by Galatanu et al.^21^

For each concentration, at temperatures higher than the transition temperature, *T*_H-ISO_, SAXS curves (Figure 3 and Figure S1) present two broad peaks instead of the three sharp peaks of the hexagonal phase, indicating spherical micellar aggregation. Below the transition temperature, *T*_H-ISO_, there is a stabilization of lattice spacing at a relatively constant value and a saturation of the primary peak intensity, indicating a stabilization of order in the hexagonal phase.

**Figure 4:**
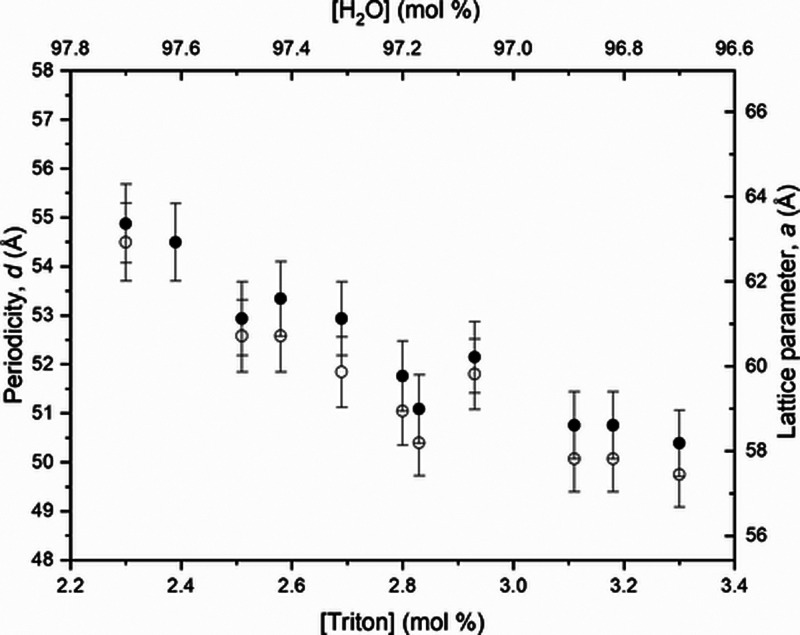
Periodicity, *d*, and lattice parameter, *a*, determined for all the nondoped samples, with [Triton X] from 2.3 to 3.3 mol %, for temperatures of *T* = 6 °C (open circles) and *T* = 23 °C (solid circles).

Periodicity, *d*, and lattice parameter, *a*, were also determined for all the samples with [Triton X] from 2.3 to 3.3 mol % for different temperatures, and the results at 6 and 23 °C are exhibited in Figure 4. The lattice parameter, *a*, and periodicity, *d*, for all measured temperatures, are shown in Tables S2 and S3, respectively, in the Supporting Information. Some sample concentrations present a rapid change in value near the transition point due to high fluctuations during the phase transition. Ahir et al.^22^ also found this behavior in their measurements due to the increase in the amplitude of the lateral thermal vibration in the transition region, causing the effective spacing to increase. Also, the intensity of the scattering peak {200} is less intense that its analog {100}, a classical Debye–Waller reduction,^22^ as shown in all graphics of Figure 3 and Figure S1.

It is interesting to notice that the periodicity and lattice parameter decrease as [Triton X] increases. This effect is more pronounced for low temperatures (far from the transition temperature, *T*_H-ISO_).

### Liquid Crystal with a Constant [Triton X] = 2.8 mol % with [DMSO] Doping from 0 to 5.0 mol %

**Figure 5:**
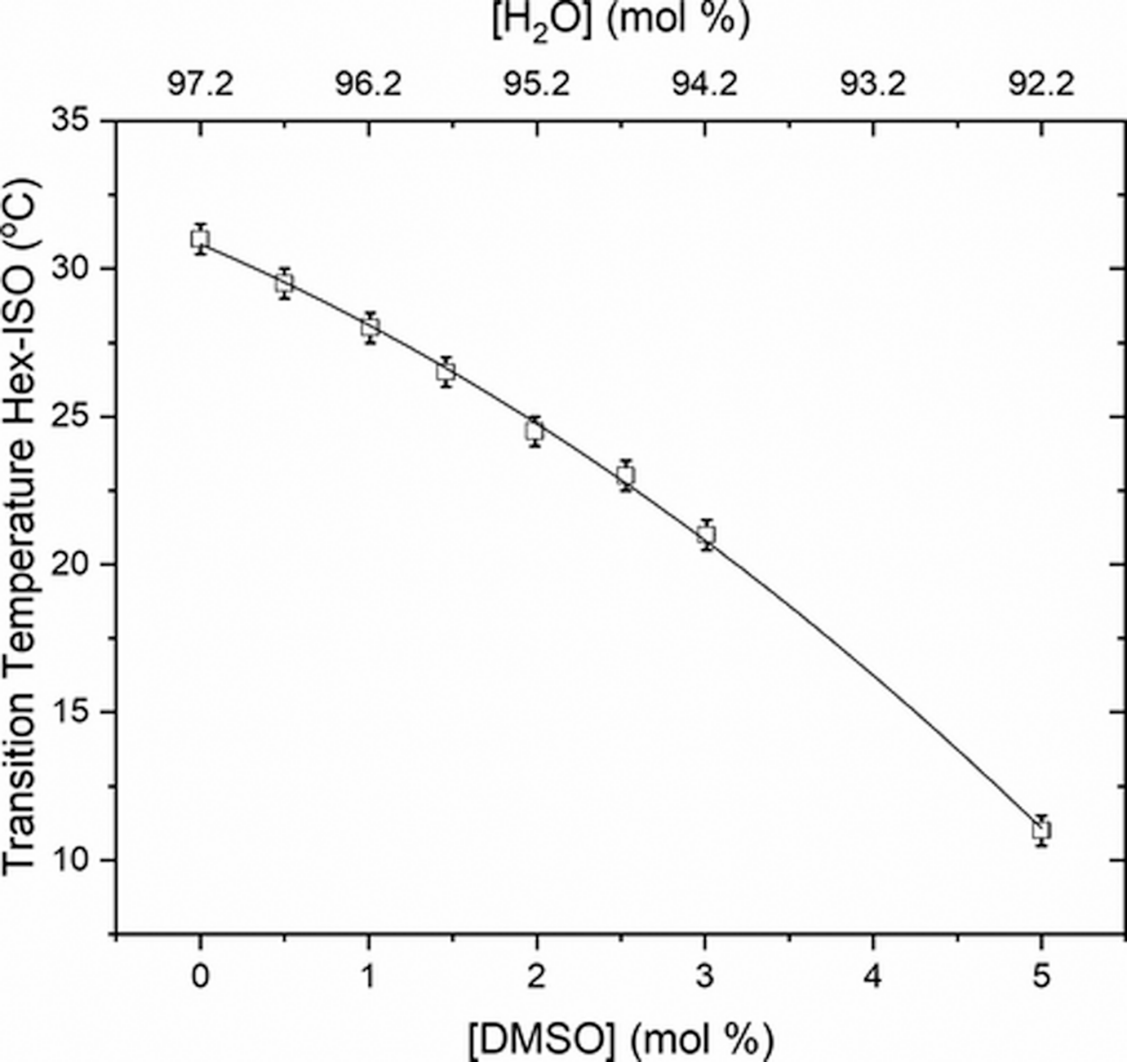
Transition temperature (squares), *T*_H-ISO_, from the hexagonal to isotropic phase for constant [Triton X] = 2.8 mol % and [DMSO] doping up to 5.0 mol %. Second-order polynomial fit (solid line).

After characterizing a hexagonal region from 2.3 to 2.7 mol % with POM and SAXS measurements, the sample with [Triton X] = 2.8 mol % and [H_2_O] = 97.2 mol % was chosen to be doped with DMSO (concentration values in Table S4 in the Supporting Information). A new set of samples were prepared with [Triton X] kept at 2.8 mol % and with tiny amounts of DMSO. Figure 5 presents the transition temperatures, *T*_H-ISO_, for constant [Triton X] = 2.8 mol % and [DMSO] doping up to 5.0 mol %. There is a considerable decrease in the phase transition temperature, *T*_H-ISO_, reducing the hexagonal region in the phase diagram and completely extinguishing the hexagonal mesophase for [DMSO] over 5.0 mol %. The best fit for the *T*_H-ISO_ data (*R*^2^ = 0.99904) is a second-order polynomial function, *T*_H-ISO_ = 30.83(18) – 2.42(17)[DMSO] – 0.31(3)[DMSO]^2^; the constant term is the transition temperature without any doping.

**Figure 6:**
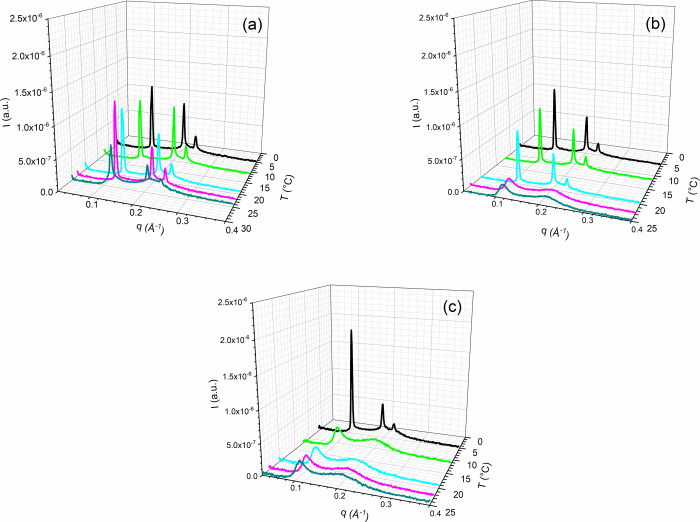
Diffraction peaks of the hexagonal phases at different temperatures for [DMSO] from 0 to 5.0 mol % in the 2.8 mol % samples. (a) [DMSO] = 0.5 mol %, (b) [DMSO] = 2.0 mol %, and (c) [DMSO] = 5.0 mol %.

SAXS measurements were performed through a range of temperatures, and Figure 6 shows some diffractograms obtained at different temperatures for [DMSO] from 0 to 5.0 mol % in the 2.8 mol % samples. The complete series is shown in Figure S2 in the Supporting Information. With increasing [DMSO], diffraction peaks disappeared, for higher temperatures.

**Figure 7:**
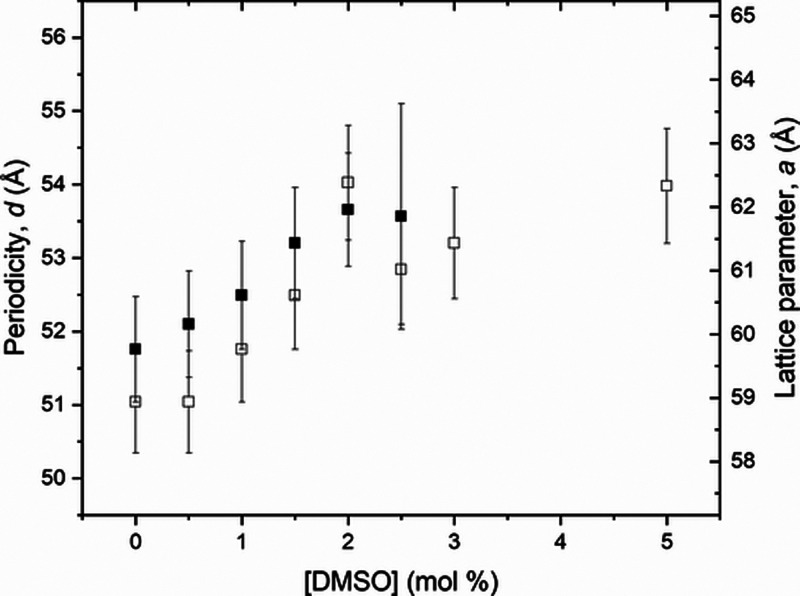
Periodicity, *d*, and lattice parameter, *a*, determined for all DMSO doping, from 0 up to 5.0 mol %, in the samples with [Triton X] = 2.8 mol %, for temperatures of *T* = 6 °C (open squares) and *T* = 23 °C (solid squares).

From the graphics, Figure S2, the periodicity, *d*, and the lattice parameter, *a*, were calculated for all samples in different temperatures; Figure 7 presents these results at 6 and 23 °C.

Values calculated for the lattice parameter, *a*, are all measured at different temperatures in Table S5; results for periodicity, *d*, are in Table S6 in the Supporting Information.

Observing Figure 7 and Tables S5 and S6, it is clear that the addition of DMSO promotes an increase in the lattice parameter, *a*, and in the periodicity, *d*. Comparing Figures 4 and 7, the rise in the periodicity values, *d*, is the same from 51 to 55 Å, even with adding DMSO to the 50/50 wt % sample ([Triton X] = 2.8 mol %) or increasing the water proportion from 97.2 to 97.7 mol % (Triton X decreasing from 2.8 to 2.3 mol %) in the nondoped samples. This fact makes us believe that DMSO is in the water region of the hexagonal phase since DMSO is miscible in water.

On the other hand, comparing the Hex-ISO phase transition temperatures, *T*_H-ISO_, in Figures 2 and 5, there is a decrease of approximately 20 °C in transition temperature when adding DMSO to the 2.8 mol % sample. In contrast, the transition temperature variation is less than 1 °C when the water proportion varies from 97.2 to 97.7 mol % in the nondoped samples.

So, adding DMSO to the 2.8 mol % sample promotes the same increase in periodicity, *d*, and lattice parameter, *a*, as increasing the water proportion but a significant difference in transition temperatures. What is the role of DMSO in the liquid crystalline phase? Two more DMSO dopings were done to answer that question.

### Liquid Crystal with [Triton X] from 2.8 to 3.3 mol %, with [DMSO] Doping from 0 to 5.0 mol %, Increasing Proportional to [Triton X]

[Triton X] was changed from 2.8 to 3.3 mol %, while the doping with DMSO varied from 0 to 5.0 mol % [DMSO], proportional to [Triton X] (concentration values in Table S7 in the Supporting Information). Again, samples were investigated using POM and SAXS measurements.

**Figure 8:**
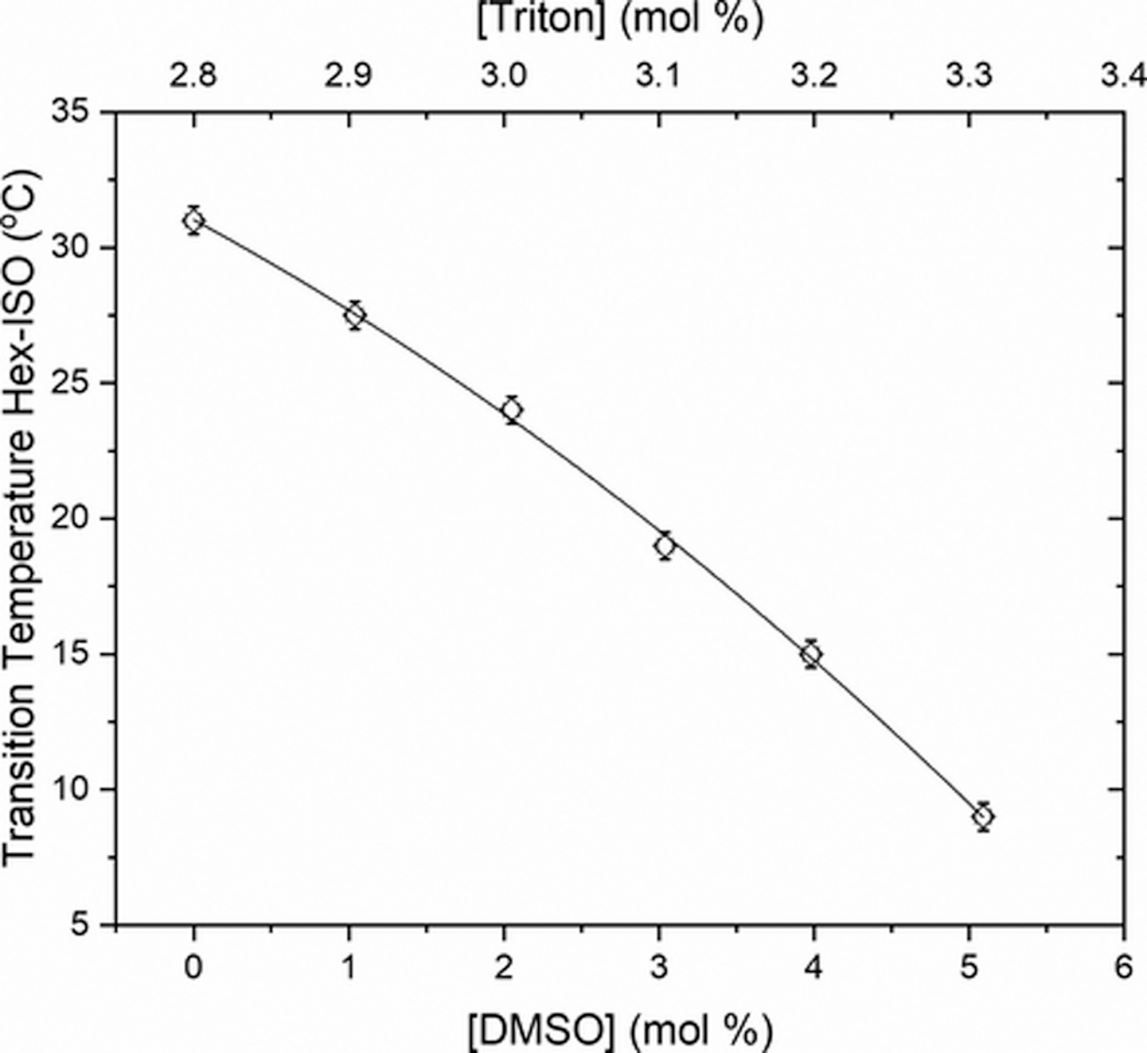
Transition temperature (diamonds), *T*_H-ISO_, from the hexagonal to isotropic phase as a function of [DMSO], in the samples with Triton X concentrations from 2.8 to 3.3 mol % and DMSO doping from 0 to 5.0 mol %. Second-order polynomial fit (solid line).

Figure 8 presents the transition temperature, *T*_H-ISO_*,* for [Triton X] from 2.8 to 3.3 mol % and [DMSO] doping up to 5.0 mol %. Again, there is a considerable decrease in phase transition temperature compared to Figure 2, reducing the hexagonal region in the phase diagram and extinguishing the hexagonal phase completely for [DMSO] over 5.0 mol %. A second-order polynomial function is the best fit (*R*^2^ = 0.99918) for the data, *T*_H-ISO_ = 31.03(28) – 3.09(25)[DMSO] – 0.24(5)[DMSO]^2^; the constant term is the transition temperature without any doping.

As can be seen in Figure 8, as [DMSO] increases from 0 to 5.0 mol %, the transition temperature decreases from 31 to 9 °C. Comparing the behavior of *T*_H-ISO_ in Figure 8 with that in Figure 5, the temperature change is almost equal, indicating that the [DMSO] is responsible for the changing temperature interval for the existing hexagonal phase.

SAXS measurements were performed over a range of temperatures. Diffractograms obtained at different temperatures for [DMSO] from 0 to 5.0 mol % in the samples with [Triton X] from 2.8 to 3.3 mol % are presented in Figure S3 in the Supporting Information. Increasing [DMSO] is increased in the same way as in Figure 6, and diffraction peaks disappeared for the higher temperatures.

**Figure 9:**
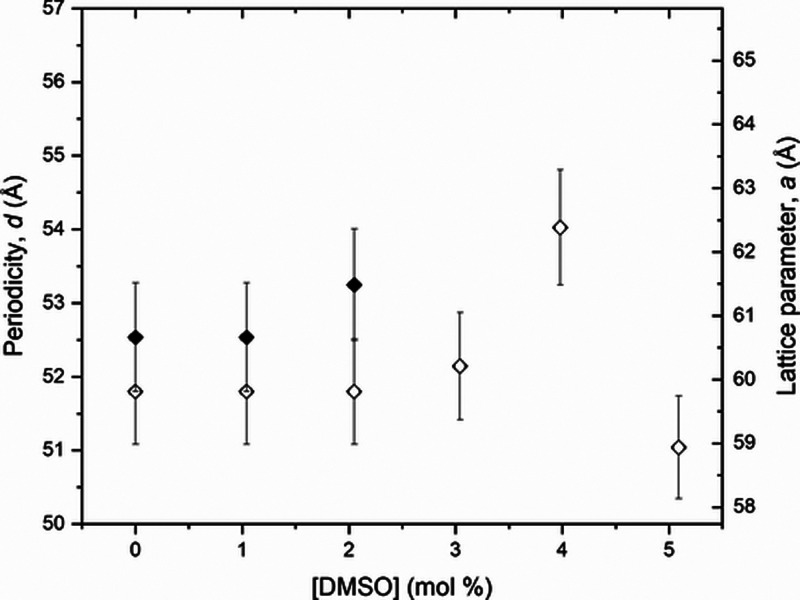
Periodicity, *d*, and lattice parameter, *a*, determined for all [DMSO] doping, from 0 up to 5.0 mol %, in the samples with [Triton X] from 2.8 to 3.3 mol %, for temperatures of *T* = 6 °C (open diamonds) and *T* = 23 °C (solid diamonds).

From these SAXS results, the periodicity, *d*, and the lattice parameter, *a*, were calculated for all samples in different temperatures; Figure 9 presents these results at 6 and 23 °C.

Values calculated for the lattice parameter, *a*, for all different measured temperatures are in Table S8; results for periodicity, *d*, are in Table S9 in the Supporting Information.

### Liquid Crystal with [Triton X] from 2.3 to 2.7 mol %, with [DMSO] Doping from 0 to 5.0 mol %, Increasing Inversely Proportional to [Triton X]

Another doping was done by varying [Triton X] from 2.3 to 2.7 mol % and [DMSO] from 0 to 5.0 mol %, with [DMSO] increasing inversely proportional to [Triton X] (concentration values in Table S10 in the Supporting Information). All samples were investigated using POM and SAXS measurements.

**Figure 10:**
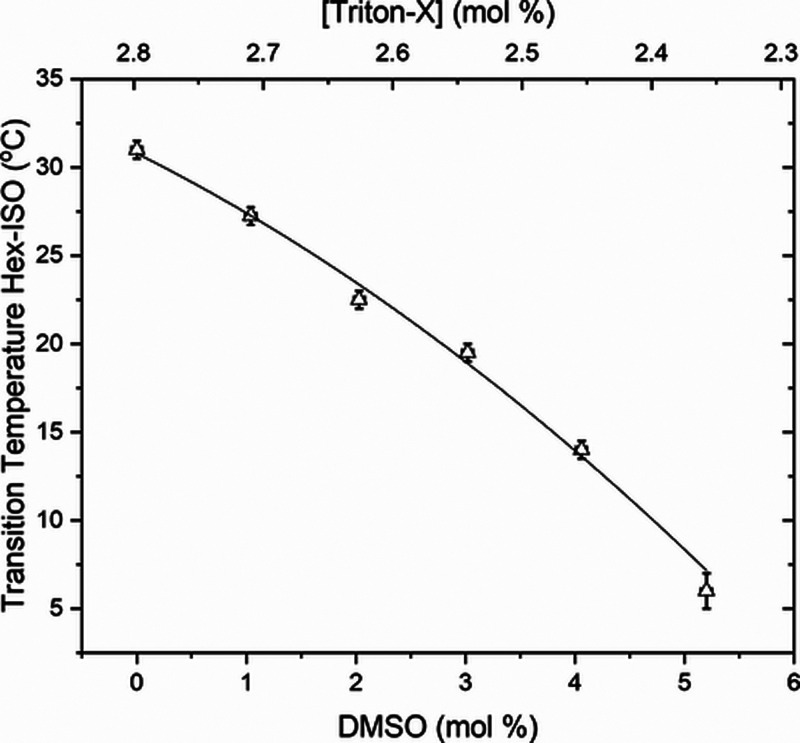
Transition temperature (triangles), *T*_H-ISO_, from the hexagonal to isotropic phase as a function of [DMSO], for [Triton X] from 2.3 to 2.8 mol % and [DMSO] from 0 to 5.0 mol %. Second-order polynomial fit (solid line).

Figure 10 presents the transition temperature *T*_H-ISO_ for [Triton X] from 2.3 to 2.7 mol % and [DMSO] from 0 to 5.0 mol %. Again, there is a considerable decrease in phase transition temperature compared to Figure 2, reducing the hexagonal region in the phase diagram and completely extinguishing the hexagonal phase for [DMSO] over 5.0 mol %. A second-order polynomial function is the best fit (*R*^2^ = 0.99336) for the data, *T*_H-ISO_ = 30.8(7) – 3.1(7)[DMSO] – 0.28(14)[DMSO]^2^; the constant term is the transition temperature without any doping.

SAXS measurements were performed through a range of temperatures. The diffractograms obtained for [DMSO] from 0 to 5.0 mol % in the [Triton X] from 2.3 to 2.7 mol % samples are presented in Figure S4 in the Supporting Information. In the same way as in Figure 6, diffraction peaks disappear at increasing [DMSO], for the higher temperatures.

**Figure 11:**
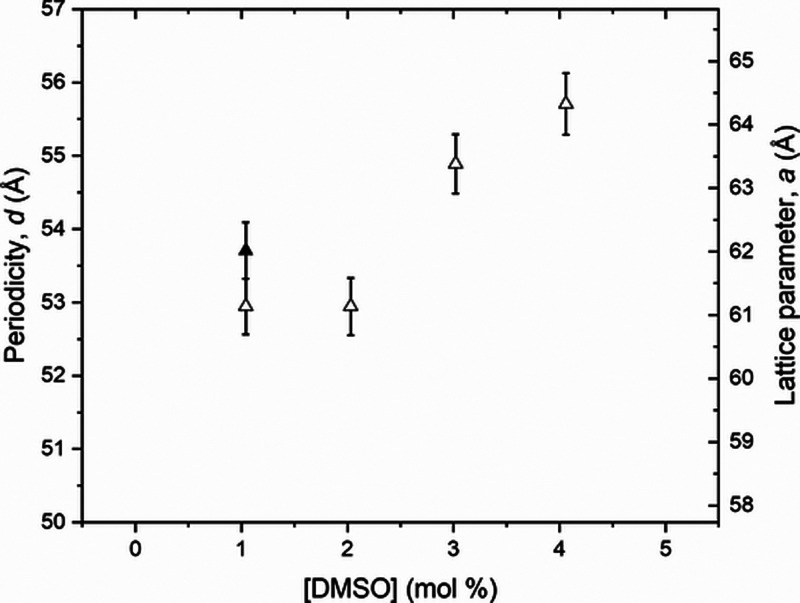
Periodicity, *d*, and lattice parameter, *a*, determined for all DMSO doping, from 0 up to 5.0 mol %, in the samples with [Triton X] from 2.3 to 2.7 mol %, for temperatures *T* = 6 °C (open triangles) and *T* = 23 °C (solid triangle).

From these SAXS results, the periodicity, *d*, and the lattice parameter, *a*, were calculated for all samples in different temperatures; Figure 11 presents these results at 6 and 23 °C.

Values calculated for the lattice parameter, *a*, for all different measured temperatures are in Table S11; results for periodicity, *d*, are in Table S12 in the Supporting Information.

**Figure 12:**
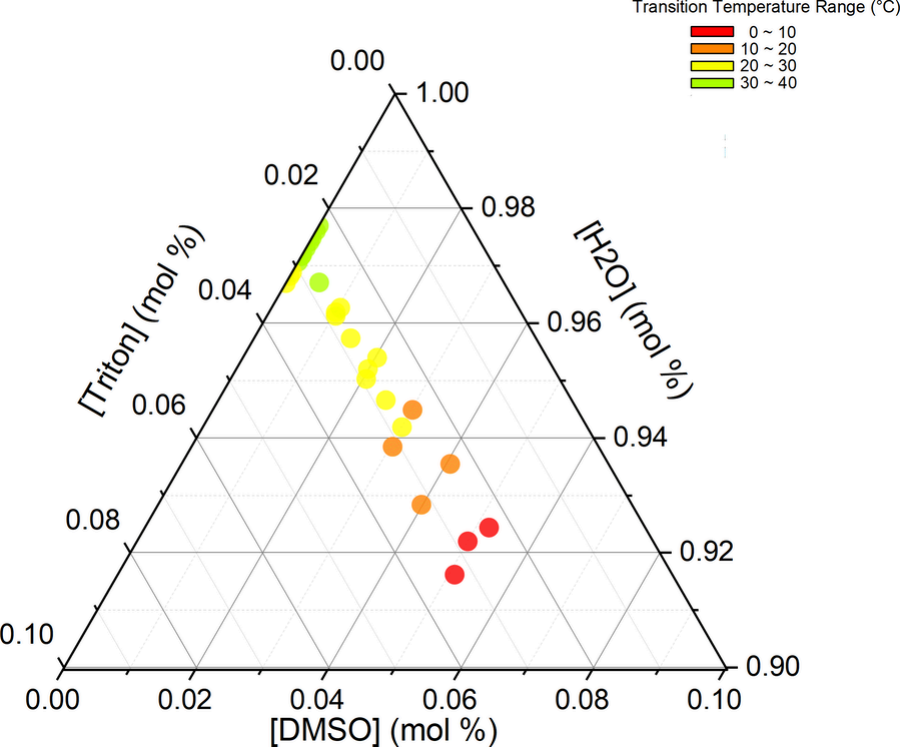
Ternary diagram with all sample concentrations and phase transition temperatures ranging from the hexagonal to isotropic phase.

With the results from Figures 2, 5, 8, and 10, we construct Figure 12, a ternary diagram with all sample concentrations utilized and the phase transition range of temperatures from the hexagonal to isotropic phase. For samples without DMSO, phase transition temperatures are kept almost constant and start decreasing with [H_2_O] decreasing (or [Triton X] increasing). When comparing samples doped with DMSO, phase transition temperatures decrease with increasing [DMSO] (or decreasing [H_2_O]). How do we decide which one, [H_2_O] or [DMSO], is responsible for reducing the phase transition temperature? The phase diagrams for Triton X and water from references^20,22^ show that a hexagonal phase exists between 35 and 60 wt % Triton X, which is the same between 40 and 65 wt % H_2_O; this concentration is equal to say between 95.8 and 98.5 mol % H_2_O. In the ternary diagram from Figure 12, we can see the phase transition temperature range from the hexagonal to isotropic phase until 92 mol % H_2_O, which is out of the range of the hexagonal phase of the binary system. Considering [H_2_O+DMSO], it is inside the hexagonal range. So, is DMSO acting as water?

As seen in Figure 5, the first point in the graph corresponds to a sample without DMSO, with [H_2_O] = 97.2 mol %. The last point corresponds to a sample with [H_2_O] = 92.2 mol % and [DMSO] = 5.0 mol %, resulting in [H_2_O+DMSO] = 97.2 mol %. The total solvent amount is the same, but the phase transition temperatures are different. In addition, doping with more than 5.0 mol % [DMSO] causes the hexagonal phase to disappear. So, the DMSO has its own role in the system.

**Figure 13:**
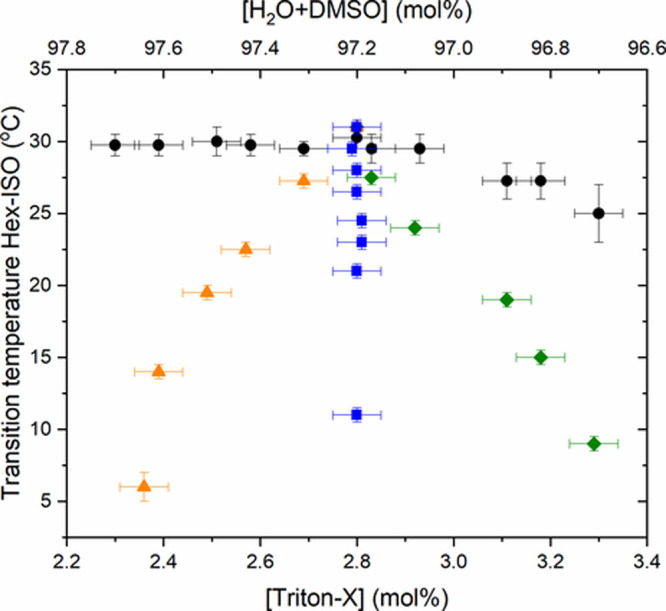
Phase transition temperatures *T*_H-ISO_ for all sample concentrations as a function of [Triton X] and [H_2_O+DMSO]. (black circles) no DMSO doping; (blue squares) [Triton X] = 2.8 mol % and [DMSO] from 0 to 5.0 mol %; (green diamonds) [Triton X] from 2.8 to 3.3 mol % and [DMSO] from 0 to 5.0 mol %; (orange triangles) [Triton X] from 2.3 to 2.8 mol % and [DMSO] from 0 to 5.0 mol %.

Figure 13 corroborates those results. It is possible to see that by keeping constant [Triton X] (square points) and adding DMSO to the sample, the phase transition temperature *T*_H-ISO_ decreases. Changing the amount of Triton X by water (triangle points) phase transition temperature *T*_H-ISO_ varied, but the temperature range is much lower than that of Triton X (diamond points). Each triangle and diamond point has a corresponding circle point with the same [Triton X] and the same [H_2_O+DMSO], but the phase transition temperature of each pair is different, and one more time, [DMSO] is responsible for decreasing the phase transition temperature.

When comparing the polynomial curve fits of the second order in Figures 5, 8, and 10, all of them have precisely the same behavior of the hexagonal to isotropic phase transition temperature as a function of [DMSO], the constant term being the hexagonal to isotropic phase transition temperature without DMSO, and the coefficients of the first and second degree are negatives, which means the decreasing rate of *T*_H-ISO_ with DMSO. A weighted average result is *T*_H-ISO_ = 30.88(15) – 2.65(14)[DMSO] – 0.291(25)[DMSO]^2^, and the hexagonal phase would completely disappear at [DMSO] = 0.6 mol %. Even though we do not have a phenomenological theory to explain second-order polynomial behavior, it is possible to conclude that DMSO has a particular role in the system, differing from the water role, and the primary role of DMSO is in decreasing the transition temperature, *T*_H-ISO_, reducing the range of temperatures of the hexagonal phase.

The values of the hexagonal lattice parameter, *a*, and periodicity, *d,* increase with DMSO doping and are compatible between them when compared to Figures 4, 7, 9, and 11. With these values and with the volume fraction of the apolar domains, ϕ, the radius of the apolar domains,^25^*R*, in the hexagonal structures can be calculated as
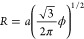


**Figure 14:**
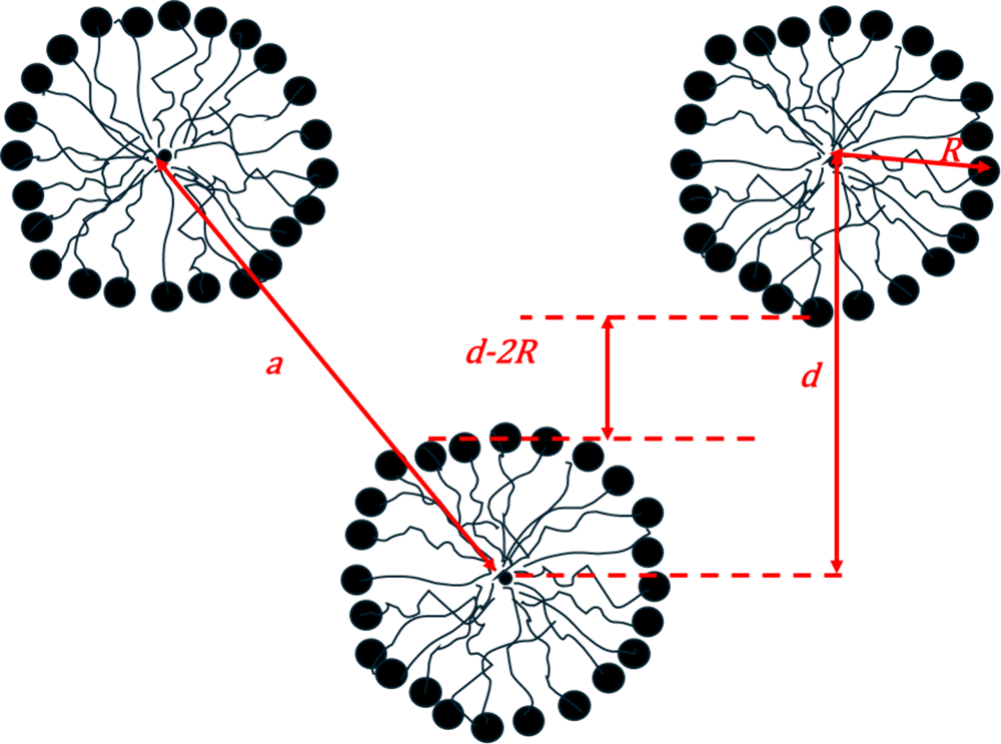
Scheme of a hexagonal structure, where *a* is the lattice parameter, *d* is the periodicity, *R* is the radius of the apolar domains, and *d*-*2R* is the width of the water channels between the hexagonally arranged micelles.

According to the hexagonal structure scheme in Figure 14, it is possible to vary the periodicity, *d*, and the width of the water channels between the hexagonally arranged micelles, *d-2R*, even with keeping *d* constant. For this reason, these parameters were determined for all configurations of [DMSO] and [Triton X].

**Figure 15:**
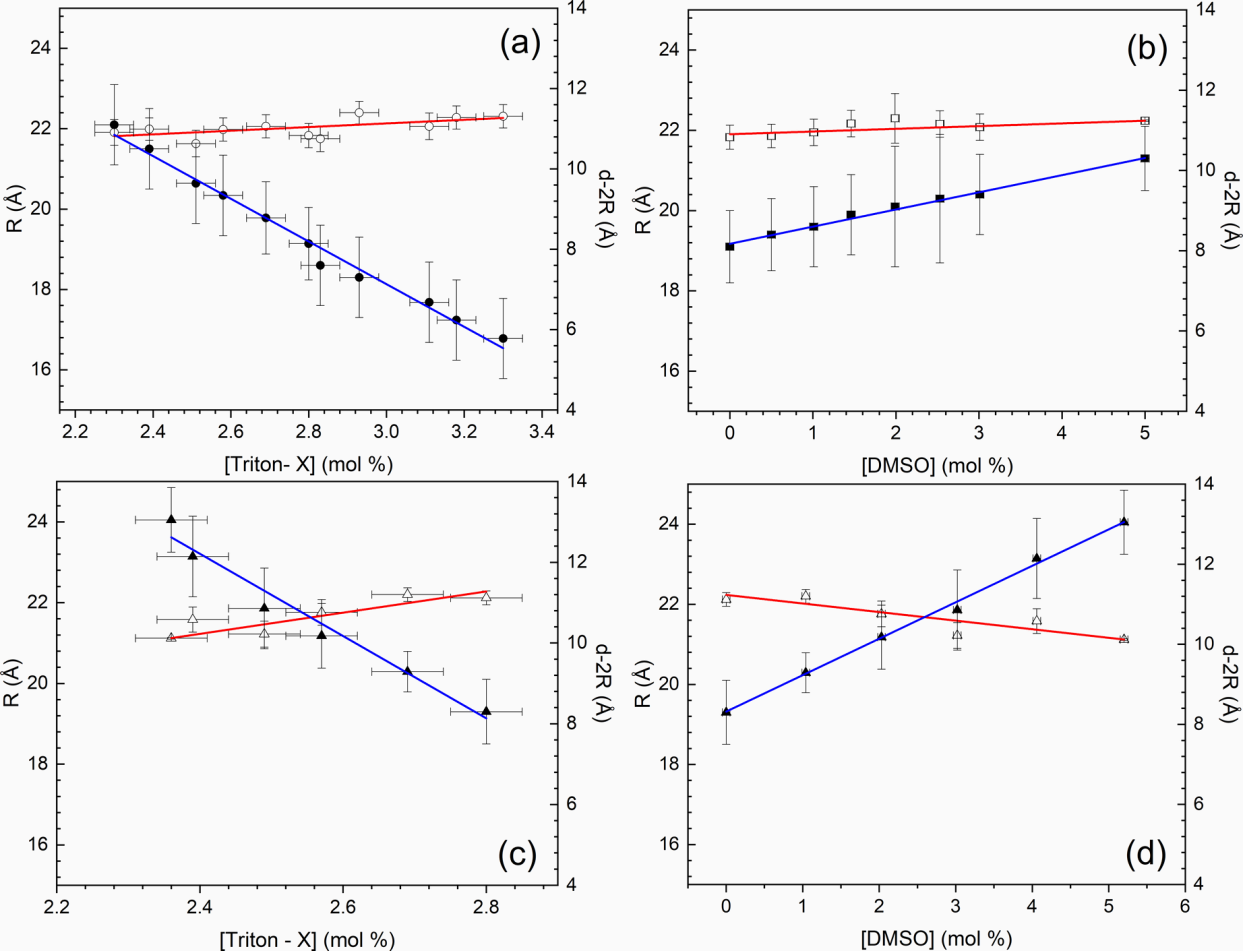
Open symbols refer to the radius of the apolar domains, *R* (left-side scale), and closed symbols refer to the width of the water channels between the hexagonally arranged micelles, *d-2R* (right-side scale). (a) No DMSO doping, [Triton X] from 2.2 to 3.4 mol %; (b) [Triton X] = 2.8 mol % and [DMSO] up to 5 mol %; (c,d) [Triton X] from 2.3 to 2.8 mol % and [DMSO] up to 5 mol %.

In Figure 15a, the binary system Triton X/water, without DMSO addition, keeps constant (slope = 0.45 ± 0.29 Å/mol %, *R*^2^ = 0.385948) the radius of the apolar domains, *R*, for [Triton X] from 2.2 to 3.4 mol %, while the width of the water channels between the hexagonally arranged micelles, *d-2R,* decreases (slope = −5.3 ± 1.0 Å/mol %, *R*^2^ = 0.986958) with [Triton X] (or increase with [H_2_O]). It is an indication that the water is out of the cylindrical micelles.

In Figure 15b, [Triton X] = 2.8 mol % and *R* were kept constant (slope = 0.07 ± 0.03 Å/mol %, *R*^2^ = 0.860395); instead, the width of the water channels between the hexagonally arranged micelles, *d-2R,* increased (slope = 0.43 ± 0.20 Å/mol %, *R*^2^ = 0.998206) with [DMSO] doping. Considering that the distance *d-2R* refers to the space outside of the cylindrical micelles, the result indicates that DMSO is in the polar domain with the water.

For [DMSO] increasing proportional to the [Triton X] increase, there is no significant effect, and the linear fittings resulted in very large uncertainties; this can be due to a “competition” between polar and apolar regions' enlargement. In agreement with Figure 15b, Figure 15c,d presents a small decrease (slope = −0.214 ± 0.025 Å/mol %, *R*^2^ = 0.955470) in the radius of the apolar domains, *R* values, with [DMSO] increasing (the same with [Triton X] decreasing), while the width of the water channels between the hexagonally arranged micelles, *d-2R,* increased (slope = 0.91 ± 0.18 Å/mol %, *R*^2^ = 0.997509) practically linearly with [DMSO] doping.

With the results of Figure 15, it can be stated that DMSO is in the polar domain since it is a polar solvent, just like water. The values obtained for the radius of the apolar domains, *R*, are of the order of 22 (1) Å, which is in good agreement with previous papers for spherical micelles of Triton X in aqueous solution^27^ or in an ethylene glycol–water mixture.^28^

## Conclusions

This article studied the role of DMSO in a binary system of Triton X and water in the hexagonal mesophase. With this purpose, four series of samples were done, each of them keeping constant a different parameter, which means no DMSO doping with [Triton X] from 2.3 to 3.3 mol %, DMSO doping from 0 to 5.0 mol % with [Triton X] = 2.8 mol %, DMSO doping from 0 to 5.0 mol % with [Triton X] from 2.8 to 3.3 mol %, and DMSO doping from 0 to 5.0 mol % with [Triton X] from 2.8 to 2.3 mol %. The series of samples were studied with POM and SAXS measurements, and the effect of DMSO on the binary system Triton X/water was determined.

For the intervals of concentrations studied in this paper, Triton X molecules aggregate in cylindrical micelles in a hexagonal 2D arrangement, while DMSO and water molecules form the polar external region. Once DMSO molecules are in the polar region, there is a strong interaction between DMSO and water, with DMSO molecules distorting the hydrogen bond network of water. This provides greater structuring of water, with a well-defined hydration structure around the oxygen atom of DMSO, which can establish strong hydrogen bonds with the surrounding water molecules;^7^ due to this, the cohesive force decreases in the binary mixture Triton X/water, and surfactant molecules are more soluble in a mixed solvent.^9,10^ This can be thought of as if it were necessary to increase the Triton X concentration to maintain the stability of the hexagonal phase. As a result, there is a great destabilization of the hexagonal arrangement with increasing [DMSO], reducing the transition temperature, *T*_H-ISO_, from the hexagonal to isotropic phase until the hexagonal phase completely disappears when [DMSO] = 5.0 mol %. Even though phase transition temperature, *T*_H-ISO_, can change with [Triton X] and [H_2_O] (Figure 2), the decrease in *T*_H-ISO_ due to DMSO doping follows exactly the same behavior, depending just on [DMSO]: *T*_H-ISO_ = 30.88(15) – 2.65(14)[DMSO] – 0.291(25)[DMSO]^2^. The destabilization of the hexagonal arrangement with increasing [DMSO] is clearly seen in the series of diffractograms in the Supporting Information, with the loss of the third diffraction peak and width enlargement of the first and second one, losing the typical hexagonal relationships .

The slight increase in periodicity and lattice parameter with increasing [DMSO] is due to the insertion of DMSO molecules in the water region, causing a greater distance between the cylindrical micelles, while the radius of the apolar domains remained constant at 22 (1) Å.

This work opens new possibilities to manipulate the phase transition temperature in the binary system Triton X/water by just adding a small quantity of DMSO, keeping constant the radius of the apolar domains and with an increase of about 5 Å in the water channels between the hexagonally arranged micelles.

## Supporting Information Available

Sample concentration tables, order parameter and lattice parameter tables for all samples and measured temperatures, and diffractogram graphics for all samples and measured temperatures (PDF)

## ABBREVIATIONS

*a*: hexagonal lattice parameter
*d*: hexagonal periodicity
*d*-*2R*: width of the water channels between the hexagonally arranged micelles
DMSO: dimethyl sulfoxide
[DMSO]: DMSO concentration
ϕ: volume fraction of the apolar domains
[H_2_O]: H_2_O concentration
Hex: hexagonal phase
*I*: intensity
ISO: isotropic phase
Triton X: Triton X-100 polyethylene glycol *tert*-octylphenyl ether
[Triton X]: Triton X concentration
POM: polarized optical microscopy
*q*: scattering vector modulus
*R*: radius of the apolar domains
*R*^2^: *R*-squared or the coefficient of determination
SAXS: small-angle X-ray scattering
SI: Supporting Information
*T*: temperature
*T*_H-ISO_: phase transition temperature of hexagonal to isotropic
wt: weight
