# Supplementary material for: Effect of DMSO Addition on the Hexagonal Phase of the System Triton X/Water
Source: Langmuir. 2024 Oct 11;40(42):21985–94. doi: 10.1021/acs.langmuir.4c01937 (PMC11500494; doi:10.1021/acs.langmuir.4c01937)
Supplement: Supplementary file 1 — la4c01937_si_001.pdf [file la4c01937_si_001.pdf]

Supporting Information

Effect of DMSO addition in hexagonal phases of the system Triton X/water

Leila T. Thieghi and Sarah I.P.M.N. Alves*

Instituto de Ciências Ambientais, Químicas e Farmacêuticas, Universidade Federal de São Paulo, Diadema – SP, Brasil– Postal Code 09913-030

* leila.thieghi@unifesp.br

Number of pages: 13

Number of figures: 4

Number of schemes:0

Number of tables: 12

Table of contents:

Table S1: Concentrations of Triton X and water for the non-dopped samples with [Triton X] from 2.3 mol % to 3.3 mol %_____ S3

Table S2: Lattice parameter, *a*, for all non-doped samples, with [Triton X] from 2.3 mol % to 3.3 mol %, for different temperatures. Values with a grey background correspond to temperatures above the transition temperature, TH-ISO. _____ S3

Table S3: Periodicity, *d*, for all samples for [Triton X] from 2.3 mol % to 3.3 mol % for different temperatures. Values with a grey background correspond to temperatures above the transition temperature, TH-ISO._____ S4

Table S4: Triton X, water, and DMSO concentrations for samples with constant [Triton X] = 2.8 mol %._____ S4

Table S5: Lattice parameter, *a*, for all [DMSO] doping, from 0 up to 5.0 mol %, in samples with [Triton X] = 2.8 mol % for different temperatures. Values with a grey background correspond to temperatures above the transition temperature. _____ S5

Table S6: Periodicity, *d*, for all [DMSO] doping, from 0 up to 5.0 mol %, in the samples with [Triton X] = 2.8 mol % for different temperatures. Values with a grey background correspond to temperatures above the transition temperature. _____ S5

Table S7: Triton X, water, and DMSO concentrations for the samples with [Triton X] from 2.8 mol % to 3.3 mol %._____ S6

Table S8: Lattice parameter, *a*, for all [DMSO] doping, from 0 up to 5.0 mol %, in the samples with [Triton X] from 2.8 mol % to 3.3 mol %, for different temperatures. Values with a grey background correspond to temperatures above the transition temperature. S6

Table S9: Periodicity, *d*, for all [DMSO] doping, from 0 up to 5.0 mol %, in the samples with [Triton X] from 2.8 mol % to 3.3 mol %, for different temperatures. S7

Table S10: Triton X, water, and DMSO concentrations for the samples with [Triton X] from 2.3 mol % to 2.8 mol %. S7

Table S11: Lattice parameter, *a*, for all DMSO doping, from 0 up to 5.0 mol %, in the samples with [Triton X] from 2.3 mol % to 2.8 mol %, for different temperatures. Values with a grey background correspond to temperatures above the transition temperature. S8

Table S12: Periodicity, *d*, for all DMSO doping, from 0 up to 5.0 mol %, in the samples with Triton X concentration from 2.3 mol % to 2.8 mol %, for different temperatures. Values with a grey background correspond to temperatures above the transition temperature. S8

Figure S1: Three orders of diffraction peaks obtained at different temperatures for the samples with [Triton X] from 2.3 mol % to 3.3 mol %. (a) [Triton X] = 2.3 mol %; (b) [Triton X] = 2.4 mol %; (c) [Triton X] = 2.5 mol %; (d) [Triton X] = 2.6 mol %; (e) [Triton X] = 2.7 mol %; (f) [Triton X] = 2.8 mol %; (g) [Triton X] = 2.9 mol %; (h) [Triton X] = 3.0 mol %; (i) [Triton X] = 3.1 mol %; (j) [Triton X] = 3.2 mol %; (k) [Triton X] = 3.3 mol %. S9

Figure S2: Diffraction peaks of the hexagonal phases obtained at different temperatures for [DMSO] from 0 to 5.0 mol % in the [Triton X] = 2.8 mol % samples. (a) [DMSO] = 0.5 mol %; (b) [DMSO] = 1.0 mol %; (c) [DMSO] = 1.5 mol %; (d) [DMSO] = 2.0 mol %;(e) [DMSO] = 2.5 mol %;(f) [DMSO] = 3.0 mol %; (g) [DMSO] = 5.0 mol %. S11

Figure S3: Three orders of diffraction peaks obtained at different temperatures for the samples with [Triton X] from 2.8 mol % to 3.3 mol %, with [DMSO] doping from 0 to 5.0 mol %. (a) [DMSO] = 1.0 mol % and [Triton X] = 2.9 mol %; (b) [DMSO] = 2.0 mol % and [Triton X] = 3.0 mol %; (c) [DMSO] = 3.0 mol and [Triton X] = 3.1 mol %; (d) [DMSO] = 4.0 mol % and [Triton X] = 3.2 mol % (e) [DMSO] = 5.0 mol % and [Triton X] = 3.3 mol %. S12

Figure S4: Three orders of diffraction peaks obtained at different temperatures for the [Triton X] from 2.3 mol % to 2.8 mol %, with [DMSO] doping from 0 to 5.0 mol %. (a) [DMSO] = 1.0 mol % and [Triton X] = 2.7 mol %; (b) [DMSO] = 2.0 mol % and [Triton X] = 2.6 mol %; (c) [DMSO] = 3.0 mol % and [Triton X] = 2.5 mol %; (d) [DMSO] = 4.0 mol % and [Triton X] = 2.4 mol % (e) [DMSO] = 5.0 mol % and [Triton X] = 2.3 mol %. S13

Table S1: Concentrations of Triton X and water for the non-dopped samples with [Triton X] from 2.3 mol % to 3.3 mol %.

**[Triton X] (wt %)**	**[H_2O] (wt %)**	**[Triton X] (mol %)**	**[H_2O] (mol %)**
45.01	54.99	2.30	97.70
45.97	54.03	2.39	97.61
47.16	52.84	2.51	97.49
47.94	52.06	2.58	97.42
49.02	50.98	2.69	97.31
50.00	50.00	2.80	97.2
51.00	49.00	2.83	97.17
52.00	48.00	2.93	97.07
53.00	47.00	3.11	96.89
54.00	46.00	3.18	96.82
55.00	45.00	3.30	96.70

Table S2: Lattice parameter, *a*, for all non-doped samples, with [Triton X] from 2.3 mol % to 3.3 mol %, for different temperatures. Values with a grey background correspond to temperatures above the transition temperature, $T_{H\text{-}ISO}$.

**[Triton X] (mol %) / T(°C)**	**2.30**	**2.39**	**2.51**	**2.58**	**2.69**	**2.80**	**2.83**	**2.93**	**3.11**	**3.18**	**3.30**
	***a* (Å)**										
**6**	62.9(8)		60.7(7)	60.7(7)	59.9(7)	58.9(7)	58.2(7)	59.8(7)	57.8(7)	57.8(7)	57.4(7)
**10**							59.4(7)	59.8(7)	57.8(7)	57.8(7)	57.4(7)
**12**						58.9(7)	58.2(7)	59.4(7)	58.2(7)	57.4(7)	57.4(7)
**13**	62.9(8)	62.4(8)	61.1(7)	61.1(7)	60.3(7)		59.0(7)	60.2(7)	58.2(7)	57.8(7)	57.8(7)
**20**	63.4(8)	62.4(8)	60.7(7)	61.1(7)	60.7(7)	59.3(7)	59.8(7)	60.2(7)	58.2(7)	58.2(7)	58.2(7)
**23**	63.4(8)	62.9(8)	61.1(8)	61.6(8)	61.1(7)	59.8(7)	59.0(7)	60.2(7)	58.6(7)	58.6(7)	58.2(7)
**26**	63.4(8)	62.9(8)	61.1(8)	61.6(8)	61.1(7)		59.4(7)	59.4(7)	58.6(7)	59.0(7)	59.4(7)
**30**	62.0(8)	64.2(17)	62.0(8)	62.0(8)	60.3(15)	58.9(7)	61.5(8)	60.7(7)	59.4(7)	59.0(7)	59.4(7)
**32**	62.0(8)	62.0(15)	62.0(8)	60.7(15)	61.5(15)		60.2(7)	60.7(7)	59.4(7)	59.8(7)	59.4(7)

Table S3: Periodicity, *d*, for all samples for [Triton X] from 2.3 mol % to 3.3 mol % for different temperatures. Values with a grey background correspond to temperatures above the transition temperature, $T_{H\text{-}ISO}$.

[Triton X](mol %) / T(ºC)	2.30	2.39	2.51	2.58	2.69	2.80	2.83	2.93	3.11	3.18	3.30
	d **(Å)**										
**6**	54.5(8)		52.6(7)	52.6(7)	51.8(7)	51.0(7)	50.4(7)	51.8(7)	50.1(7)	50.1(7)	49.7(7)
**10**							51.4(7)	51.8(7)	50.1(7)	50.1(7)	49.7(7)
**12**						51.0(7)	50.4(7)	51.4(7)	50.4(7)	49.7(7)	49.7(7)
**13**	54.5(8)	54.1(8)	52.9(7)	52.9(7)	52.2(7)		51.1(7)	52.1(7)	50.4(7)	50.1(7)	50.1(7)
**20**	54.9(8)	54.1(8)	52.6(7)	52.9(7)	52.6(7)	51.4(7)	51.8(7)	52.1(7)	50.4(7)	50.4(7)	50.4(7)
**23**	54.9(8)	54.5(8)	52.9(7)	53.3(8)	52.9(7)	51.8(7)	51.1(7)	52.1(7)	50.8(7)	50.8(7)	50.4(7)
**26**	54.9(8)	54.5(8)	52.9(7)	53.3(8)	52.9(7)		51.4(7)	51.4(7)	50.8(7)	51.1(7)	51.4(7)
**30**	53.7(8)	55.6(17)	53.7(8)	53.7(8)	52.2(15)	51.0(7)	53.2(8)	52.5(7)	51.4(7)	51.1(7)	51.4(7)
**32**	53.7(8)	53.7(15)	53.7(8)	52.6(8)	53.2(15)		52.1(7)	52.5(7)	51.4(7)	51.8(7)	51.4(7)

Table S4: Triton X, water, and DMSO concentrations for samples with constant [Triton X] = 2.8 mol %.

[Triton] (wt %)	[H2O] (wt %)	[DMSO] (wt %)	[Triton] (mol %)	[H_2O] (mol %)	[DMSO] (mol %)
50.00	50.00	0	2.80	97.20	0
49.52	49.37	1.11	2.79	96.71	0.50
49.14	48.64	2.22	2.80	96.19	1.01
48.78	48.04	3.18	2.80	95.74	1.46
48.42	47.29	4.29	2.81	95.20	1.99
47.95	46.63	5.41	2.81	94.66	2.53
47.57	46.05	6.38	2.80	94.19	3.01
46.05	43.68	10.27	2.80	92.20	5.00

Table S5: Lattice parameter, *a*, for all [DMSO] doping, from 0 up to 5.0 mol %, in samples with [Triton X] = 2.8 mol % for different temperatures. Values with a grey background correspond to temperatures above the transition temperature.

[DMSO] (mol %) T(°C)	0	0.5	1.0	1.5	2.0	2.5	3.0	5.0
	***a* (Å)**							
**6**	58.9(8)	58.9(8)	59.8(8)	60.6(9)	62.4(9)	61.0(9)	61.4(9)	62.3(9)
**12**	58.9(8)	59.3(8)	60.2(8)	61.0(9)	61.1(9)	61.0(9)	61.4(9)	63.3(9)
**20**	59.3(8)	60.6(8)	60.6(9)	61.0(9)	61.5(9)	61.4(9)	61.4(9)	62.3(18)
**23**	59.8(8)	60.2(8)	60.6(9)	61.4(9)	62.0(9)	61.9(18)	61.9(9)	63.3(9)
**25**	59.8(8)	59.8(8)	61.4(9)	61.4(9)	61.5(9)	61.0(9)	61.4(9)	63.3(9)

Table S6: Periodicity, *d*, for all [DMSO] doping, from 0 up to 5.0 mol %, in the samples with [Triton X] = 2.8 mol % for different temperatures. Values with a grey background correspond to temperatures above the transition temperature.

[DMSO] mol %) T(°C)	0	0.5	1.0	1.5	2.0	2.5	3.0	5.0
	***d* (Å)**							
**6**	51.0(7)	51.0(7)	51.8(7)	52.5(7)	54.0(8)	52.8(7)	53.2(8)	54.0(8)
**12**	51.0(7)	51.4(7)	52.1(7)	52.8(7)	52.9(7)	52.8(7)	53.2(8)	54.8(8)
**20**	51.4(7)	52.4(7)	52.5(7)	52.8(7)	53.2(8)	53.2(8)	53.2(8)	54.0(16)
**23**	51.8(7)	52.1(7)	52.5(7)	53.2(8)	53.7(8)	53.6(15)	53.6(8)	54.8(8)
**25**	51.8(7)	51.8(7)	53.2(8)	53.2(8)	53.2(8)	52.8(8)	53.2(8)	54.8(8)

Table S7: Triton X, water, and DMSO concentrations for the samples with [Triton X] from 2.8 mol % to 3.3 mol %.

[Triton] (wt %)	[H_2O] (wt %)	[DMSO] (wt %)	[Triton] (mol %)	[H_2O] (mol %)	[DMSO] (mol %)
50	50	0	2.8	97.2	0
49.42	48.30	2.28	2.83	96.13	1.04
49.36	46.29	4.35	2.92	95.03	2.05
50.18	43.67	6.15	3.11	93.85	3.04
50.11	42.07	7.82	3.18	92.84	3.98
50.14	40.17	9.69	3.29	91.62	5.09

Table S8: Lattice parameter, *a*, for all [DMSO] doping, from 0 up to 5.0 mol %, in the samples with [Triton X] from 2.8 mol % to 3.3 mol %, for different temperatures. Values with a grey background correspond to temperatures above the transition temperature.

[DMSO] (mol %) / T(°C)	0	1.04	2.05	3.04	3.98	5.09
	a (Å)					
**6**	59.8(8)	59.8(8)	59.8(8)	60.2(8)	62.4(9)	58.9(8)
**12**						58.9(8)
**13**	60.2(8)	60.2(8)	59.8(8)	61.1(9)	61.1(9)	
**20**	60.2(8)	60.7(9)	61.5(9)	61.5(9)	61.5(9)	59.3(8)
**23**	60.7(9)	60.7(9)	61.5(9)	62.4(9)	62.0(9)	59.8(8)
**26**	60.7(9)	61.5(9)	59.8(8)	61.1(9)	61.5(9)	
**30**	60.7(9)	62.0(9)	60.2(8)	62.0(9)	62.4(9)	
**32**	60.2(8)		61.5(9)	62.0(9)	62.0(9)	

Table S9: Periodicity, *d*, for all [DMSO] doping, from 0 up to 5.0 mol %, in the samples with [Triton X] from 2.8 mol % to 3.3 mol %, for different temperatures.

[DMSO] (mol %) / T(ºC)	0	1.04	2.05	3.04	3.98	5.09
	d (Å)					
6	51.8(7)	51.8(7)	51.8(7)	52.1(7)	54.0(8)	51.0(7)
12						51.0(7)
13	52.1(7)	52.1(7)	51.8(7)	52.9(7)	52.9(7)	
20	52.1(7)	52.5(7)	53.2(8)	53.2(8)	53.2(8)	51.4(7)
23	52.5(7)	52.5(7)	53.2(8)	54.0(8)	53.7(8)	51.8(7)
26	52.5(7)	53.2(8)	51.8(7)	52.9(7)	53.2(8)	
30	52.5(7)	53.7(8)	52.1(7)	53.7(8)	54.0(8)	
32	52.1(7)		53.2(8)	53.7(8)	53.7(8)	

Table S10: Triton X, water, and DMSO concentrations for the samples with [Triton X] from 2.3 mol % to 2.8 mol %.

[Triton] (wt %)	[H_2O] (wt %)	[DMSO] (wt %)	[Triton] (mol %)	[H_2O] (mol %)	[DMSO] (mol %)
50.00	50.00	0	2.80	97.20	0
48.10	49.58	2.32	2.69	96.27	1.04
46.16	49.29	4.55	2.57	95.40	2.03
44.52	48.73	6.75	2.49	94.50	3.02
42.72	48.20	9.08	2.39	93.55	4.06
40.91	47.86	11.23	2.36	92.44	5.20

Table S11: Lattice parameter, *a*, for all DMSO doping, from 0 up to 5.0 mol %, in the samples with [Triton X] from 2.3 mol % to 2.8 mol %, for different temperatures. Values with a grey background correspond to temperatures above the transition temperature.

[DMSO] (mol %) / T(ºC)	1.04	2.03	3.02	4.06	5.20
	***a* (Å)**				
**6**	61.1(4)	61.1(5)	63.4(5)	64.3(5)	65.8(5)
**13**	61.1(4)	61.1(5)	64.3(5)	64.8(5)	65.30(10)
**20**	61.1(4)	61.1(5)	62.9(9)	63.9(9)	64.84(10)
**23**	62.0(4)	62.0(5)	61.6(9)	63.9(9)	63.87(10)
**26**	61.6(4)	61.6(9)	62.9(9)	63.9(9)	65.30(10)
**30**	61.2(9)	61.2(9)	62.9(9)	62.9(9)	65.30(10)
**32**	61.2(9)	61.2(9)	62.9(9)	63.9(9)	65.30(10)

Table S12: Periodicity, *d*, for all DMSO doping, from 0 up to 5.0 mol %, in the samples with Triton X concentration from 2.3 mol % to 2.8 mol %, for different temperatures. Values with a grey background correspond to temperatures above the transition temperature.

[DMSO] (mols%) / T(ºC)	1.04	2.03	3.02	4.06	5.20
	***d* (Å)**				
**6**	53.0(4)	53.0(4)	54.9(4)	55.7(4)	60.0(4)
**13**	53.0(4)	53.0(4)	55.7(4)	56.2(4)	56.6(9)
**20**	53.0(4)	53.0(4)	54.5(8)	55.3(8)	56.2(8)
**23**	53.7(4)	53.7(4)	53.3(8)	55.3(8)	55.3(8)
**26**	53.3(4)	53.3(8)	54.5(8)	55.3(8)	56.6(9)
**30**	53.0(8)	53.0(8)	54.5(8)	54.5(8)	56.6(9)
**32**	53.0(8)	53.0(8)	54.5(8)	55.3(8)	56.6(9)

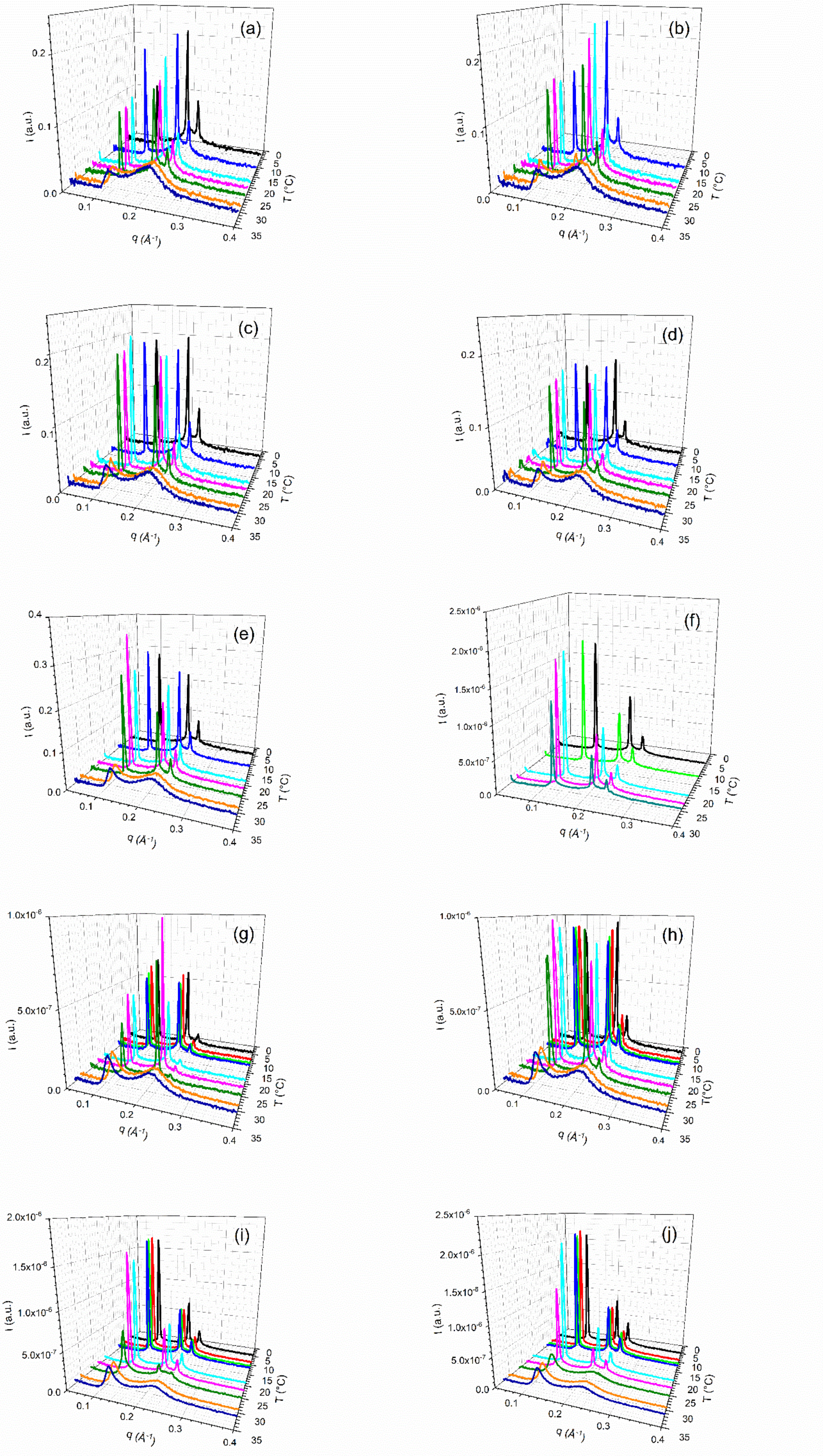
(a)
(b)
(c)
(d)
(e)
(f)
(g)
(h)
(i)
(j)
I (a.u.)
q (Å^{-1})
T (°C)

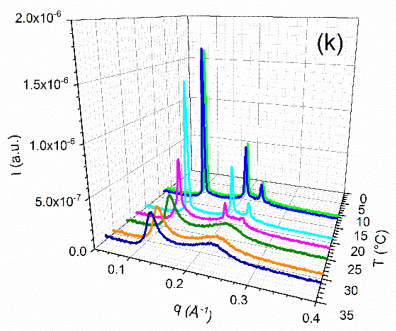

Figure S1: Three orders of diffraction peaks obtained at different temperatures for the samples with [Triton X] from 2.3 mol % to 3.3 mol %. (a) [Triton X] = 2.3 mol %; (b) [Triton X] = 2.4 mol %; (c) [Triton X] = 2.5 mol %; (d) [Triton X] = 2.6 mol %; (e) [Triton X] = 2.7 mol %; (f) [Triton X] = 2.8 mol %; (g) [Triton X] = 2.9 mol %; (h) [Triton X] = 3.0 mol %; (i) [Triton X] = 3.1 mol %; (j) [Triton X] = 3.2 mol %; (k) [Triton X] = 3.3 mol %.

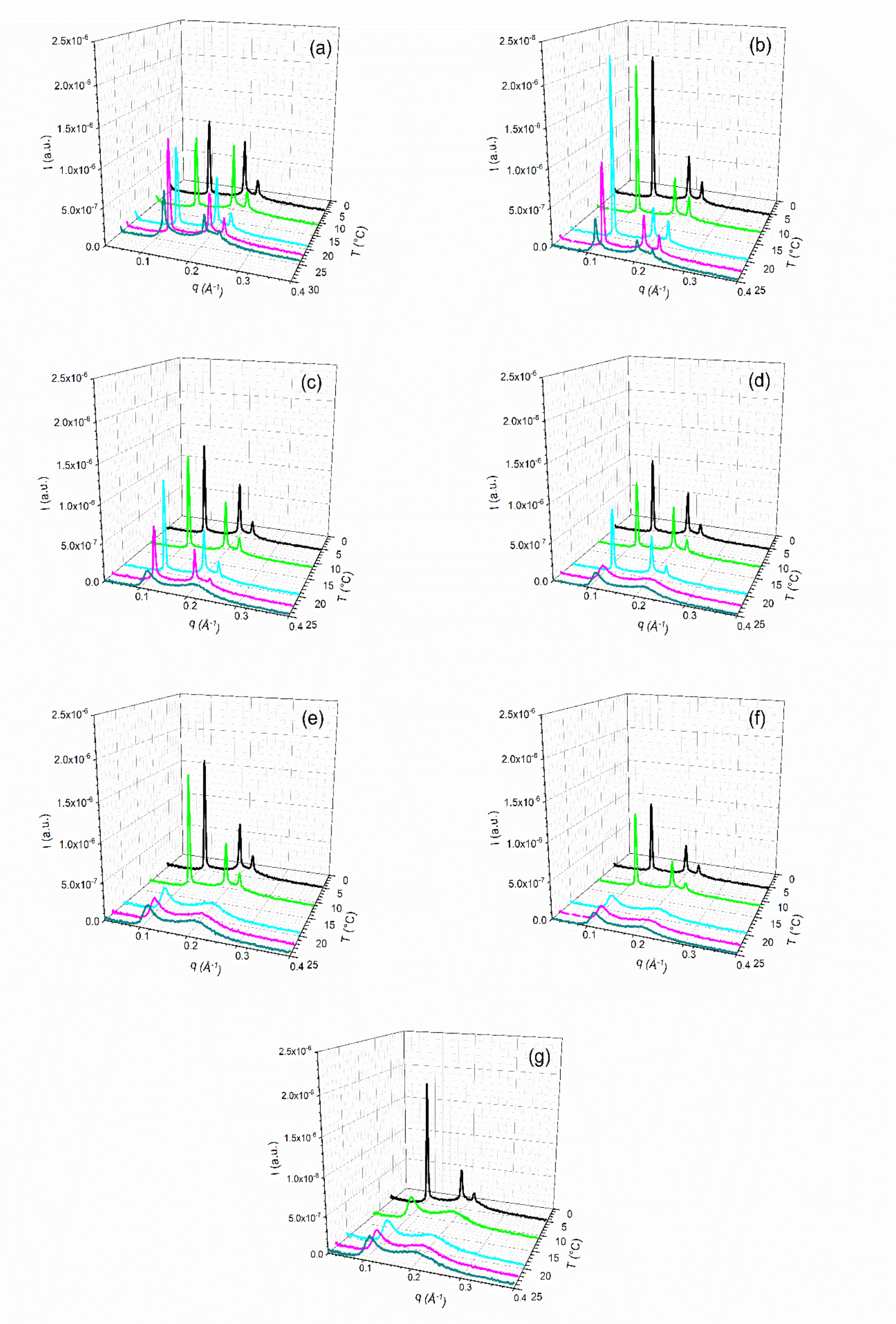

Figure S2: Diffraction peaks of the hexagonal phases obtained at different temperatures for [DMSO] from 0 to 5.0 mol % in the [Triton X] = 2.8 mol % samples. (a) [DMSO] = 0.5 mol %; (b) [DMSO] = 1.0 mol %; (c) [DMSO] = 1.5 mol %; (d) [DMSO] = 2.0 mol %;(e) [DMSO] = 2.5 mol %;(f) [DMSO] = 3.0 mol %; (g) [DMSO] = 5.0 mol %.

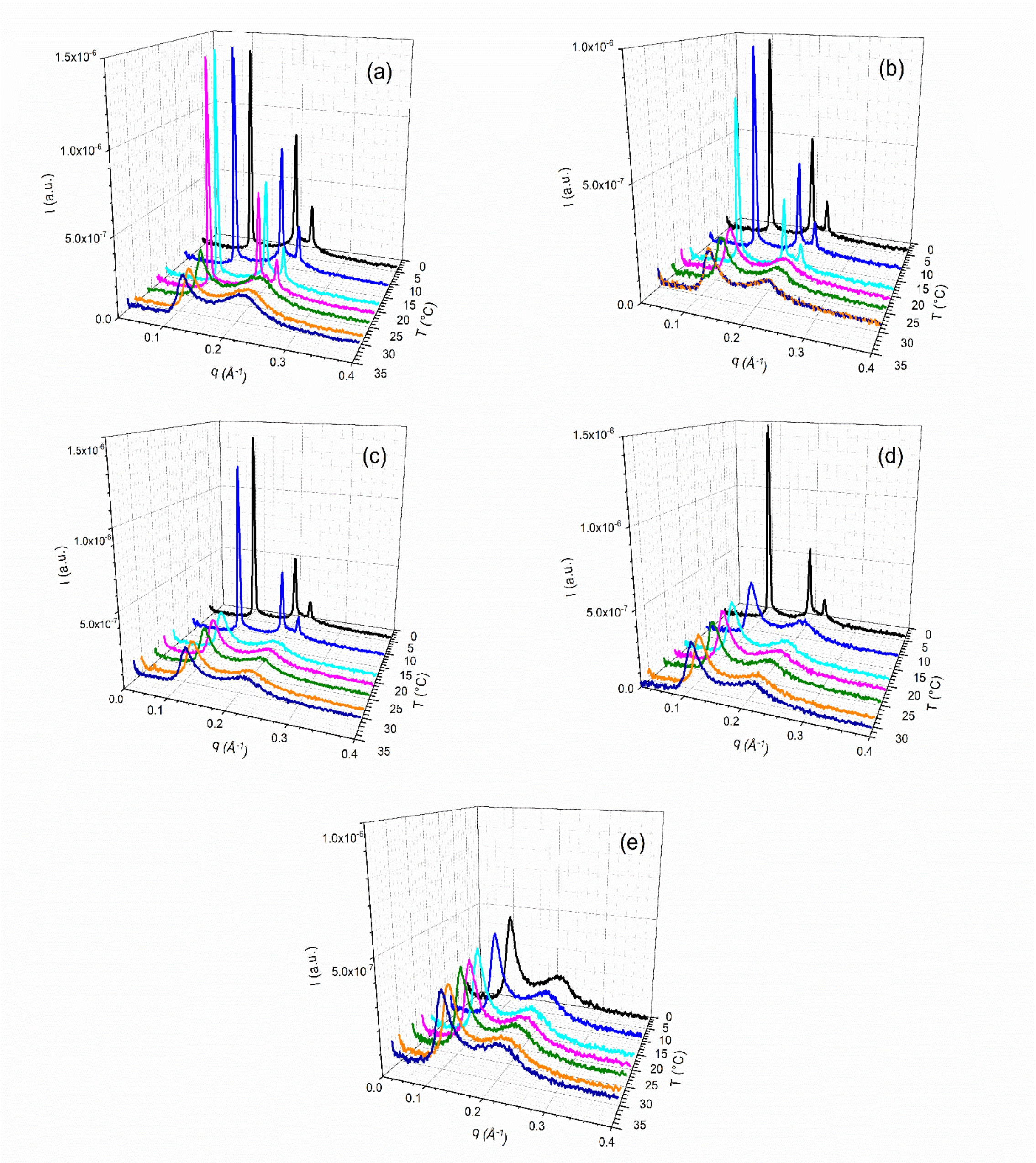

Figure S3: Three orders of diffraction peaks obtained at different temperatures for the samples with [Triton X] from 2.8 mol % to 3.3 mol %, with [DMSO] doping from 0 to 5.0 mol %. (a) [DMSO] = 1.0 mol % and [Triton X] = 2.9 mol %; (b) [DMSO] = 2.0 mol % and [Triton X] = 3.0 mol %; (c) [DMSO] = 3.0 mol and [Triton X] = 3.1 mol %; (d) [DMSO] = 4.0 mol % and [Triton X] = 3.2 mol % (e) [DMSO] = 5.0 mol % and [Triton X] = 3.3 mol %.

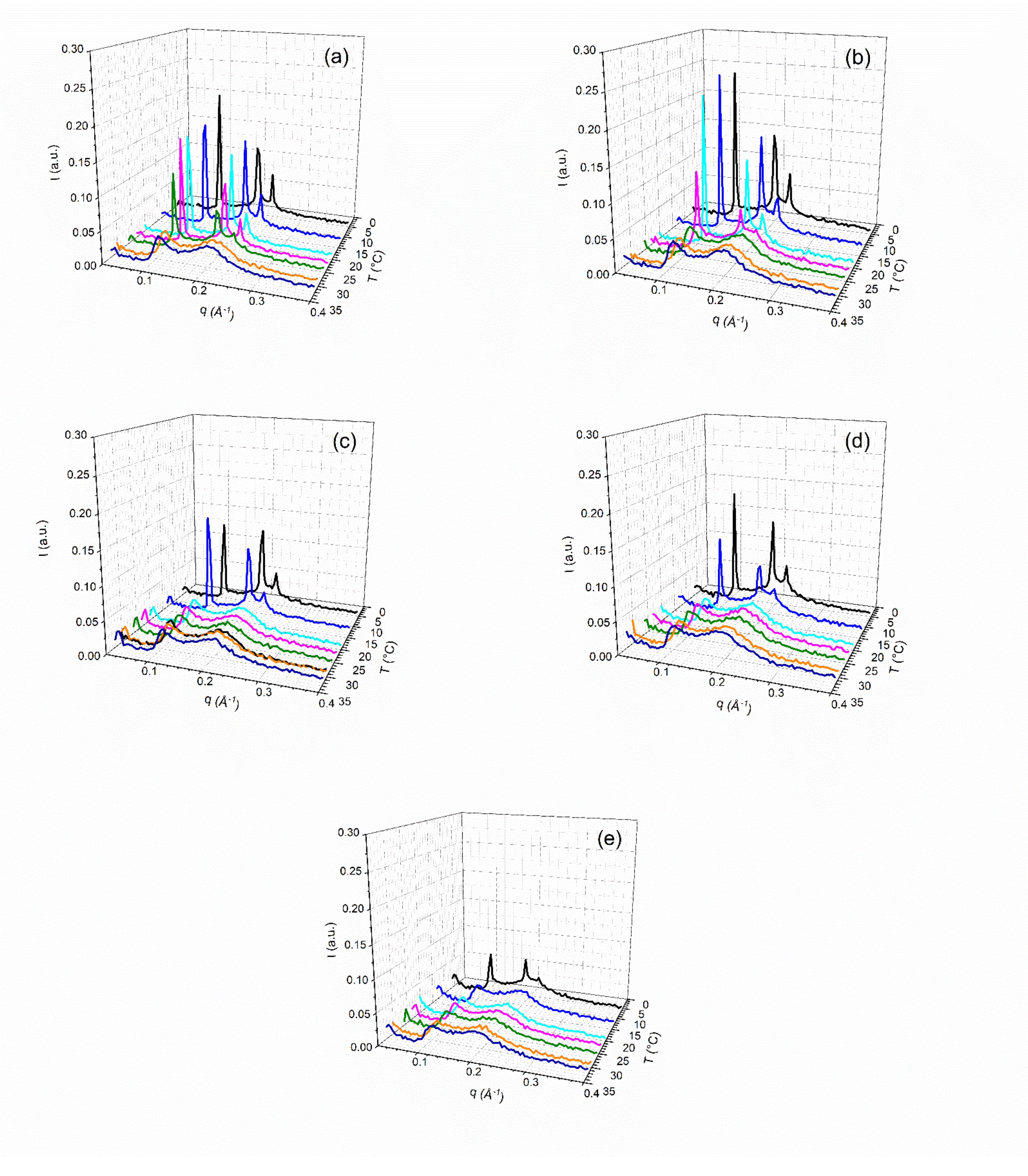

Figure S4: Three orders of diffraction peaks obtained at different temperatures for the [Triton X] from 2.3 mol % to 2.8 mol %, with [DMSO] doping from 0 to 5.0 mol %. (a) [DMSO] = 1.0 mol % and [Triton X] = 2.7 mol %; (b) [DMSO] = 2.0 mol % and [Triton X] = 2.6 mol %; (c) [DMSO] = 3.0 mol % and [Triton X] = 2.5 mol %; (d) [DMSO] = 4.0 mol % and [Triton X] = 2.4 mol % (e) [DMSO] = 5.0 mol % and [Triton X] = 2.3 mol %.
